# USP14/S100A11 axis promote colorectal cancer progression by inhibiting cell senescence

**DOI:** 10.1038/s41419-025-07724-8

**Published:** 2025-05-15

**Authors:** Yong Huang, Xiaolei Tang, Hao Xie, Zhaoying Wu, Lei Jin, Lei Zhang, Xidong Lin, Hailang Zhou, Junwei Zou

**Affiliations:** 1 Department of Gastrointestinal Surgery, The Second Affiliated Hospital of Wannan Medical College, Wuhu, Anhui China; 2 Center for Translational Medicine, The Second Affiliated Hospital of Wannan Medical College, Wuhu, Anhui China; 3 Department of Gastroenterology, The Second Affiliated Hospital of Wannan Medical College, Wuhu, Anhui China; 4 Department of Gastroenterology, Lianshui People’s Hospital Affiliated to Kangda College of Nanjing Medical University, Huai’an, Jiangsu China

**Keywords:** Colon cancer, Tumour-suppressor proteins

## Abstract

The aberrant expression of S100A11 has been identified in various malignancies but its functional roles and underlying mechanisms in colorectal cancer (CRC) have not been fully elucidated. Therefore, this study was designed to investigate the expression of S100A11 and its functional significance in CRC, indicating that S100A11 is significantly upregulated and correlates with poor survival outcomes in CRC. Functionally, S100A11 knockdown in CRC cell lines inhibited cell proliferation, invasion, and migration, leading to decreased tumour growth and metastasis in vivo. Mechanistic investigations revealed that S100A11 promotes cell proliferation and invasion by suppressing cell senescence. In addition, USP14 interacts with and mediates S100A11 deubiquitination. More importantly, the overexpression of S100A11 was able to partially counteract the reduction in cell proliferation caused by the knockdown of USP14. In summary, the novel regulatory axis involving USP14 and S100A11 modulates the malignant biological behavior of CRC cells through inhibiting cell senescence, therefore the interaction between USP14 and S100A11 represents a promising therapeutic target in CRC.

## Background

Colorectal cancer (CRC) is the third most common form of cancer and plays a major role in global mortality, with its rates of incidence and death increasing rapidly in Asia [1]. The genetic factors that contribute to the onset and development of CRC are intricate and diverse, involving somatic mutations, abnormal gene fusions, deletions or amplifications, as well as epigenetic changes and gut microbiota [2–4]. Therefore, understanding the molecular mechanisms that drive CRC initiation and progression could aid in discovering potential biomarkers for its diagnosis, prognosis, and treatment options.

In recent years, several targeted therapies have emerged as promising treatments for colorectal cancer. For instance, cetuximab and panitumumab, both monoclonal antibodies targeting the epidermal growth factor receptor (EGFR), have been widely used in patients with RAS wild-type tumours [5, 6]. Additionally, bevacizumab, an anti-VEGF antibody, has shown efficacy in combination with chemotherapy by inhibiting angiogenesis [7]. Furthermore, regorafenib, a multi-kinase inhibitor, has been approved for use in metastatic colorectal cancer following progression on standard therapies [8]. These examples underscore the diversity of molecular targets being explored in colorectal cancer treatment.

S100A11, a member of the S100 protein family, is involved in various cellular processes including cell proliferation, differentiation, and migration [9, 10]. It also plays a significant role in tumour progression by promoting epithelial-mesenchymal transition (EMT) and enhancing the metastatic potential. S100A11 is overexpressed in lung, cervical, colorectal and pancreatic cancers, where it facilitates tumour growth and invasion [11–13]. Additionally, S100A11 interacts with the receptor for advanced glycation end products (RAGE), contributing to the inflammatory tumour microenvironment and resistance to apoptosis [14].

USP14 is a deubiquitinating enzyme that regulates protein degradation by removing ubiquitin from proteasome-bound substrates, thereby influencing protein homoeostasis [15]. USP14 has been implicated in promoting tumour growth and survival by stabilising oncogenic proteins and modulating signalling pathways [16]. It is overexpressed in colorectal, breast and liver cancers, where it enhances cell proliferation and inhibits apoptosis [17–21]. Nevertheless the details of the biological effect of USP14 expression on CRC remain unclear.

In the present study, we found that S100A11 is upregulated in clinical CRC samples and correlates with advanced clinical grades of CRC. S100A11 deficiency impairs cell proliferation and invasion in CRC, inhibiting tumour growth in a xenograft model. It has also been suggested that USP14, a deubiquitination ligase, is involved in regulating S100A11 deubiquitination and inhibiting the degradation stability of S100A11, suggesting that S100A11 could be a potential diagnostic biomarker and therapeutic target for CRC.

## Results

### S100A11 is upregulated in CRC and associated with patient prognosis

**Fig. 1:**
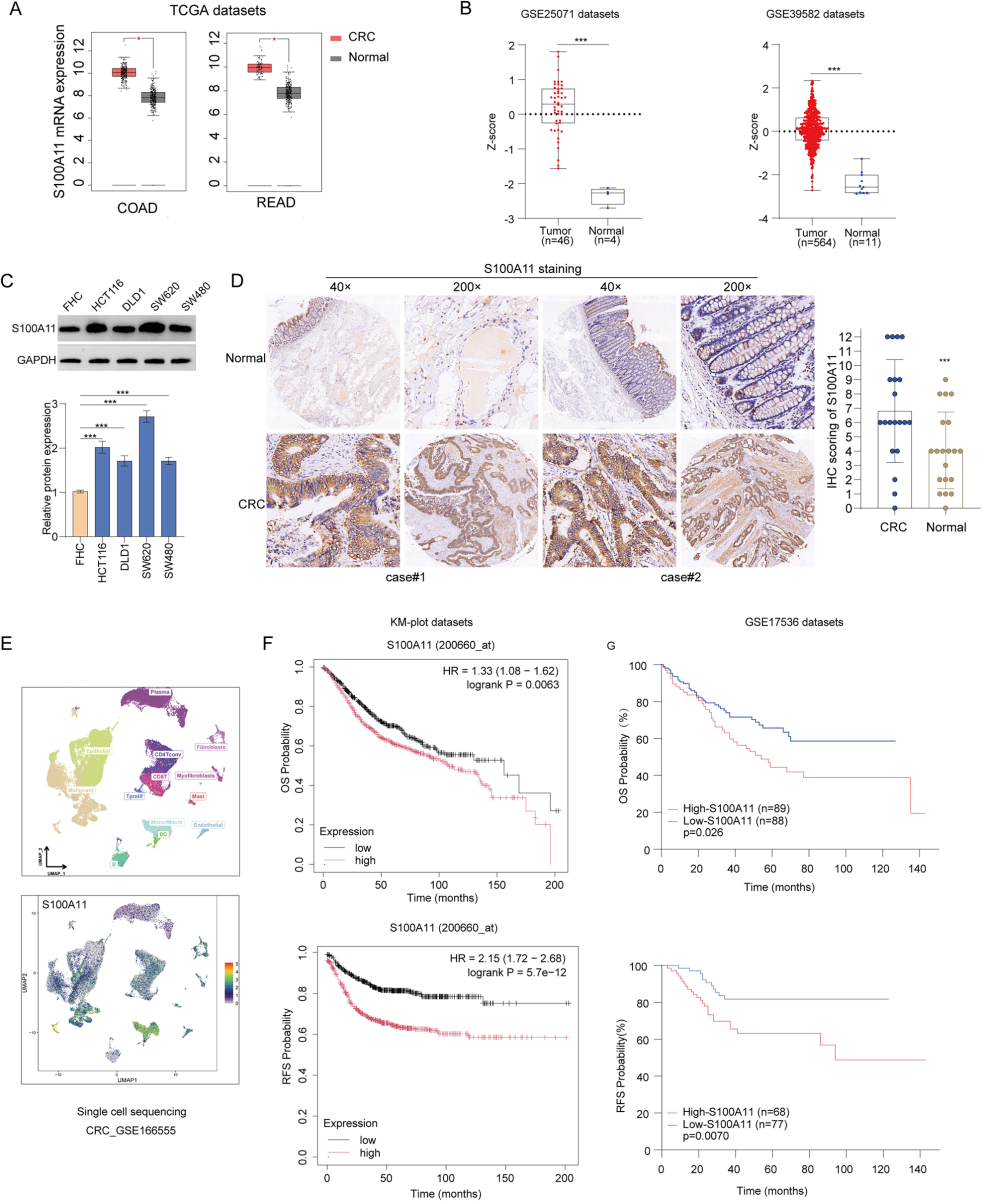
Differential S100A11 expression and prognostic significance in colorectal cancer. **A** S100A11 mRNA expression in TCGA datasets for COAD and READ. **B** S100A11 expression in GSE25071 and GSE39582 datasets. **C** Western blot analysis of S100A11 in CRC cell lines. **D** Immunohistochemical staining of S100A11 in CRC and normal tissues. **E** Single-cell sequencing showing S100A11 expression in the tumour microenvironment. **F**, **G** Kaplan–Meier survival curves for overall survival (OS) and recurrence-free survival (RFS) based on S100A11 expression in the KM-plot and GSE17536 datasets. ***p < 0.001.

S100A11 mRNA levels are upregulated in CRC tissues based on the TCGA and GEO databases (Fig. 1A, B). Likewise, S100A11 protein expression was higher in the four CRC cancer cell lines compared to the normal human epithelial cell line FHC (Fig. 1C). In addition, immunohistochemistry revealed that S100A11 protein expression was significantly higher in the primary CRC tissue samples compared to the paired normal tissue samples (Fig. 1D). Publicly available single-cell gene expression data (GSE166555) analysis reveals increased S100A11 expression in malignant cells compared to normal epithelial cells (Fig. 1E). Furthermore, the Kaplan-Meier plotter (Fig. 1F) and GEO database (Fig. 1G) revealed that high S100A11 expression is associated with inferior outcomes in CRC. Taken together, these results indicate that high S100A11 expression is associated with a poorer CRC prognosis.

### S100A11 promoted CRC proliferation and invasion

**Fig. 2:**
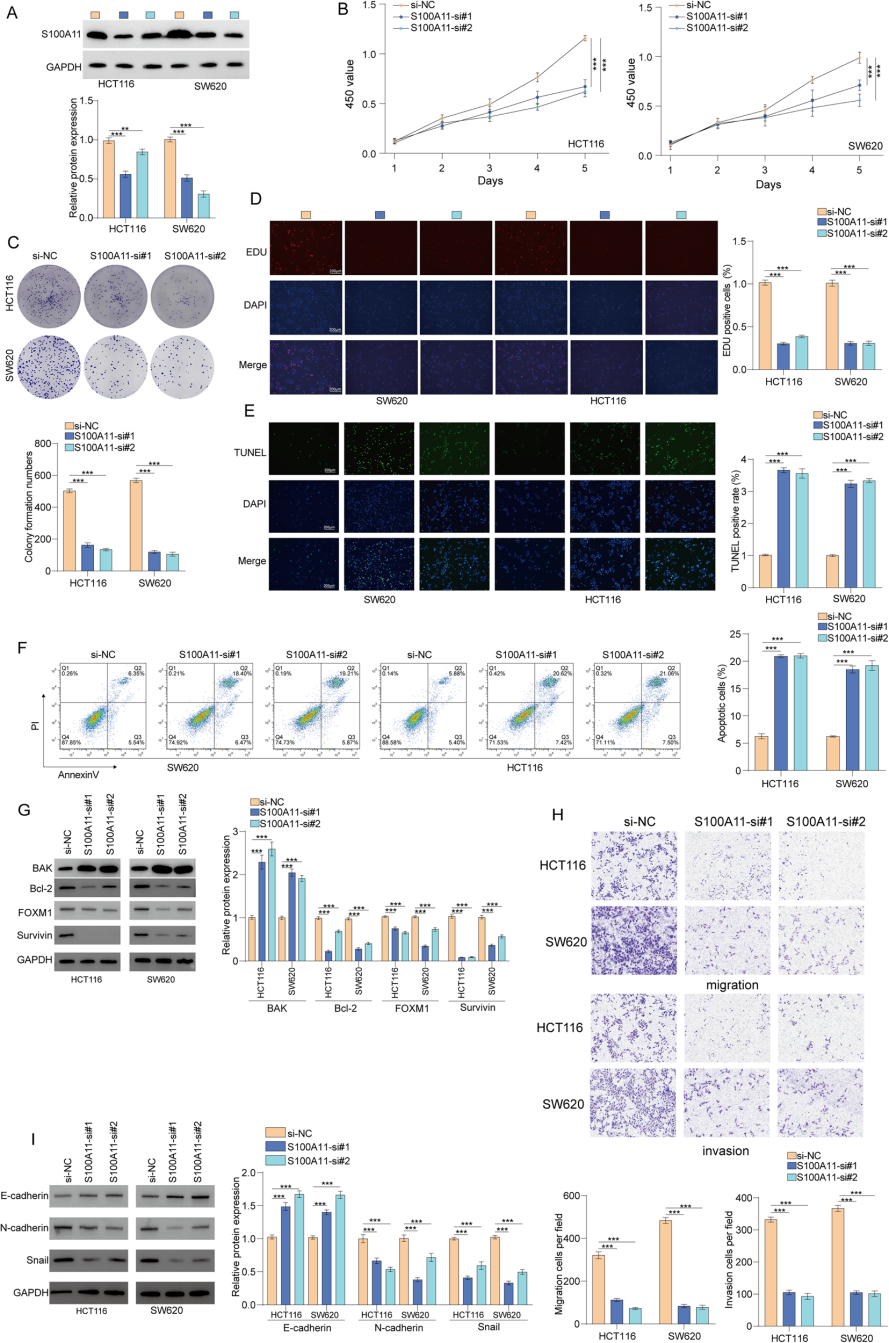
Effects of S100A11 knockdown on colorectal cancer cell proliferation, apoptosis, migration, and invasion. **A** Western blot analysis confirming S100A11 knockdown. **B** CCK-8 assay showing reduced proliferation in HCT116 and SW620 cells. **C** Colony formation assay results. **D** EdU incorporation assay indicated decreased DNA synthesis. **E**, **F** TUNEL assay and flow cytometry analysis using Annexin V/PI staining showing increased apoptosis. **G** Western blot of apoptosis-related proteins. (H) Migration and Invasion assay results. **I** Western blot analysis of EMT markers. ***p < 0.001.

Gain-of-function assays using the SW620 and HCT116 CRC cell lines were performed to determine the functional role of S100A11. Western blot analysis confirmed successful transfection (Fig. 2A). S100A11 knockdown significantly attenuated SW620 and HCT116 cell proliferation (Fig. 2B–D) and increased apoptosis (Fig. 2E, F) with upregulation of pro-apoptotic BAK and downregulation of anti-apoptotic Bcl-2, FOXM1, and survivin (Fig. 2G). S100A11 knockdown also significantly reduced cell migration and invasion (Fig. 2H). In HCT116 and SW620 cells, S100A11 knockdown significantly increased E-cadherin expression and decreased N-cadherin and Snail levels compared to the si-NC group (Fig. 2I). Ectopic S100A11 expression in SW480 cells significantly promoted cell proliferation as evidenced by colony formation, EdU, cell growth curve assays, cell migration and invasion (Supplementary Fig. S1). These results suggest that S100A11 acts as an oncogene in CRC cells.

### S100A11 promoted tumour growth and metastasis in vivo

**Fig. 3:**
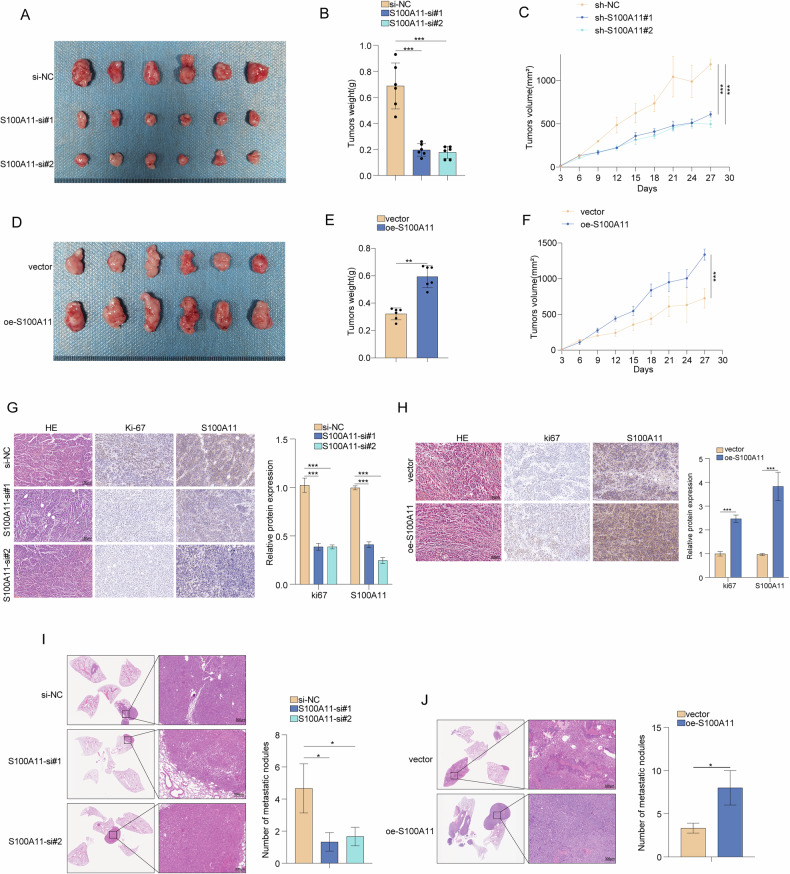
Impact of S100A11 on colorectal cancer tumour growth in vivo. **A** Representative images from S100A11 knockdown groups and control. **B** Tumour weights from the knockdown study. **C** Tumour volume growth curves for S100A11 knockdown. **D** Representative images from S100A11 overexpression and control groups. **E** Tumour weights from the overexpression study. **F** Tumour volume growth curves for S100A11 overexpression. **G** Histological analysis of Ki-67, and S100A11 staining in knockdown tumours. **H** Histological analysis in overexpression tumours. **I** Histological sections and quantification of metastatic nodules in lung tissues after S100A11 knockdown. **J** Histological sections and quantification of metastatic nodules in lung tissues following S100A11 overexpression. Representative images demonstrate increased metastasis in the oe-S100A11 group compared to the vector control. **p < 0.01, ***p < 0.001.

As shown in Fig. 3A–C, S100A11 silencing significantly decreased the growth rate and the tumour volume, implying lower proliferative ability. Conversely, S100A11 overexpression resulted in increased tumour growth, with larger and heavier tumours in the group overexpressing S100A11 compared to the vector control group. The tumour volume data confirmed the significant increase in growth rate for the S100A11 overexpression group (Fig. 3D–F). Ki-67 and S100A11 immunostaining demonstrated decreased proliferation in S100A11-silenced tumours, as indicated by lower Ki-67 expression (Fig. 3G), whereas tumours overexpressing S100A11 revealed increased Ki-67 levels indicating enhanced proliferation (Fig. 3H). Subsequently to evaluate the role of S100A11 in tumour metastasis in vivo BALB/c nude mice were injected intravenously in the tail vein with SW620 cells stably expressing either control or S100A11 shRNA. The metastatic nodules on the lung surface and in lung tissue sections of the S100A11 silencing groups were smaller than those in the control groups, whereas mice injected with cells with stable overexpression of S100A11 markedly increased tumour nodules in the lung as compared with that of controls. Taken together, S100A11 promoted metastasis in vivo (Fig. 3I, J).

### S100A11 knockdown inhibits IL-6 secretion and cellular senescence

**Fig. 4:**
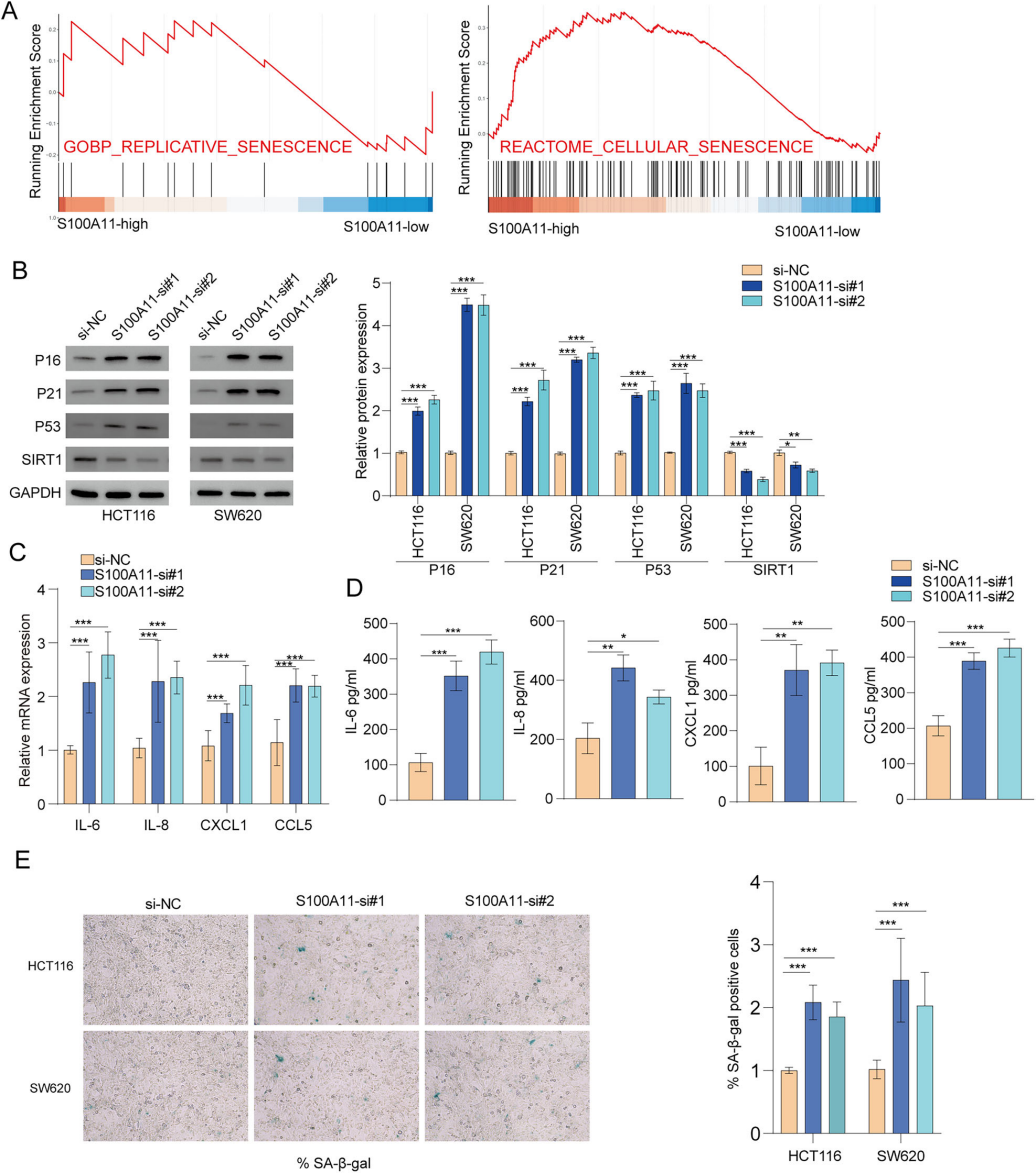
Impact of S100A11 knockdown on senescence in colorectal cancer cells. **A** GSEA plots showing enrichment of senescence-related pathways in cells with high S100A11 expression. **B** Western blot analysis of senescence and cell cycle-related proteins in HCT116 and SW620 cells after S100A11 knockdown. **C**, **D** RT-qPCR and ELISA analysis of cytokine expression levels. **E** Senescence-associated β-galactosidase staining confirmed S100A11 knockdown increased senescent cells. ***p < 0.001.

GSEA indicated that high S100A11 expression is associated with pathways related to replicative and cellular senescence (Fig. 4A), suggesting a potential role of S100A11 in modulating senescence-related processes in CRC cells. S100A11 knockdown in HCT116 and SW620 cells increased the cell cycle inhibitors P16, P21, and tumour suppressor P53, while reducing SIRT1 expression (Fig. 4B). These changes are indicative of enhanced cellular senescence upon S100A11 silencing. RT-qPCR and ELISA demonstrated that S100A11 knockdown significantly increased the expression of pro-inflammatory cytokines IL-6, IL-8, CXCL1, and CCL5, further supporting a role in cellular senescence and inflammation (Fig. 4C, D). Senescence-associated β-galactosidase staining revealed a higher percentage of senescent cells in both HCT116 and SW620 cell lines following S100A11 knockdown, confirming the induction of senescence (Fig. 4E). Collectively, these results indicate that S100A11 contributes to a senescent phenotype.

### USP14 binds to S100A11

**Fig. 5:**
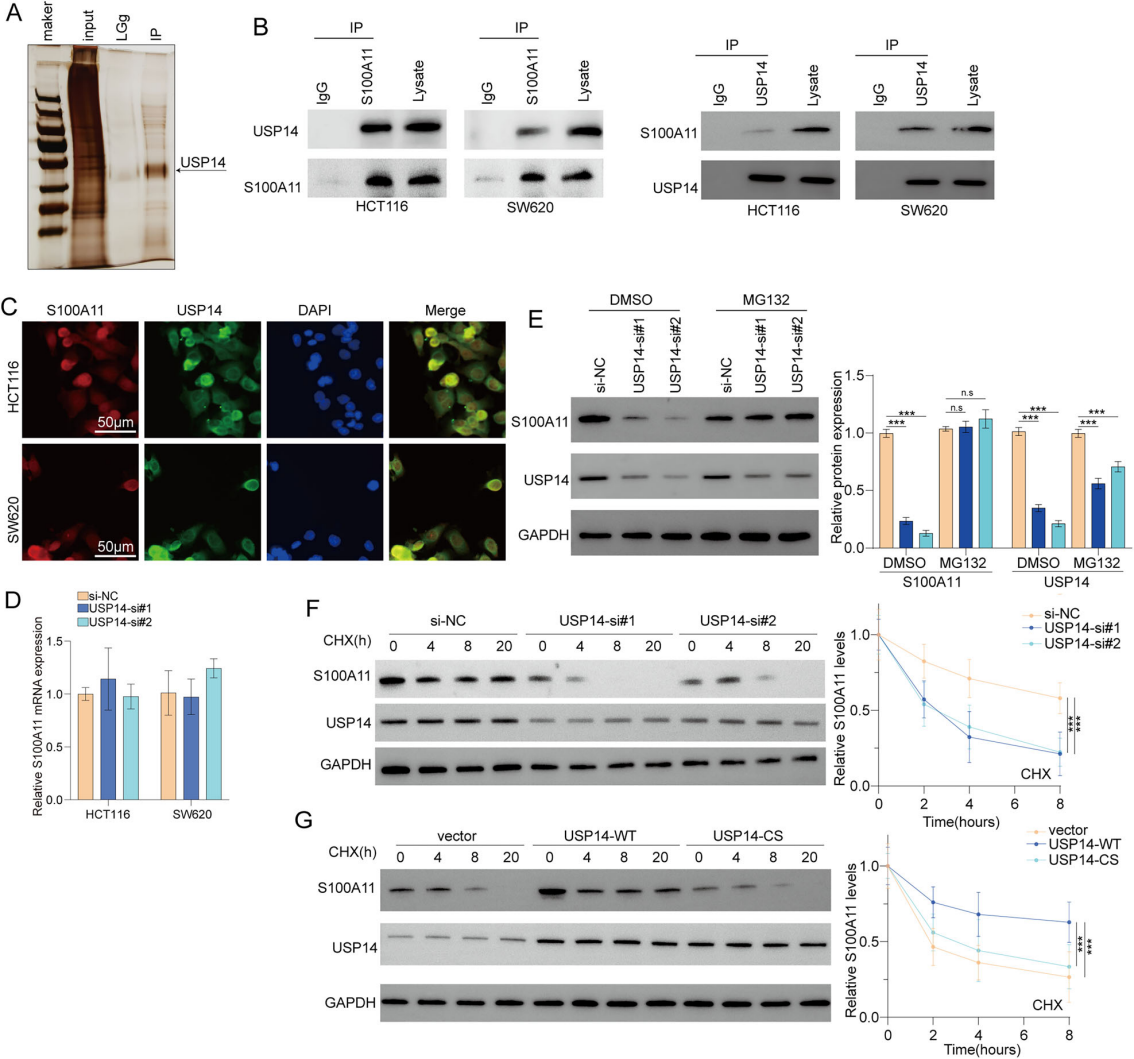
Interaction and regulation of S100A11 with USP14 in colorectal cancer cells. **A** Co-immunoprecipitation to detect USP14 interaction with S100A11. **B** Co-IP assays confirmed the interaction of S100A11 with USP14 in HCT116 and SW620 cells. **C** Immunofluorescence showing colocalisation of S100A11 and USP14. **D** USP14 levels did not significantly affect S100A11 mRNA expression. **E** Western blot showing S100A11 levels after MG132 treatment. **F** CHX chase assay demonstrating decreased S100A11 stability with USP14 knockdown. **G** CHX chase assay showed increased S100A11 stability with USP14-WT overexpression. **p < 0.01, ***p < 0.001.

USP14 was identified as a putative S100A11 binding partner (Fig. 5A). Moreover, Co-IP experiments in SW620 and HCT116 (with S100A11 overexpression) cells confirmed that USP14 binds to S100A11 (Fig. 5B) and immunofluorescence staining demonstrated the cellular colocalisation of S100A11 and USP14 (Fig. 5C). RT-qPCR analysis indicated that changes in USP14 levels did not significantly affect S100A11 mRNA expression, implying that USP14 primarily regulates S100A11 at the post-translational level (Fig. 5D). Treatment with the proteasome inhibitor MG132 resulted in the accumulation of S100A11 protein, suggesting that USP14 may prevent S100A11 degradation via the proteasome pathway (Fig. 5E). CHX chase experiments showed that USP14 knockdown significantly decreased the half-life of S100A11, indicating that USP14 stabilises S100A11 protein levels (Fig. 5F). Conversely, overexpression of wild-type USP14 (USP14-WT) increased S100A11 stability, whereas a catalytically inactive mutant (USP14-CS) did not have the same effect (Fig. 5G). In summary, USP14 interacts with and stabilises S100A11 to prevent its degradation via the proteasome pathway in CRC cells.

**Fig. 6:**
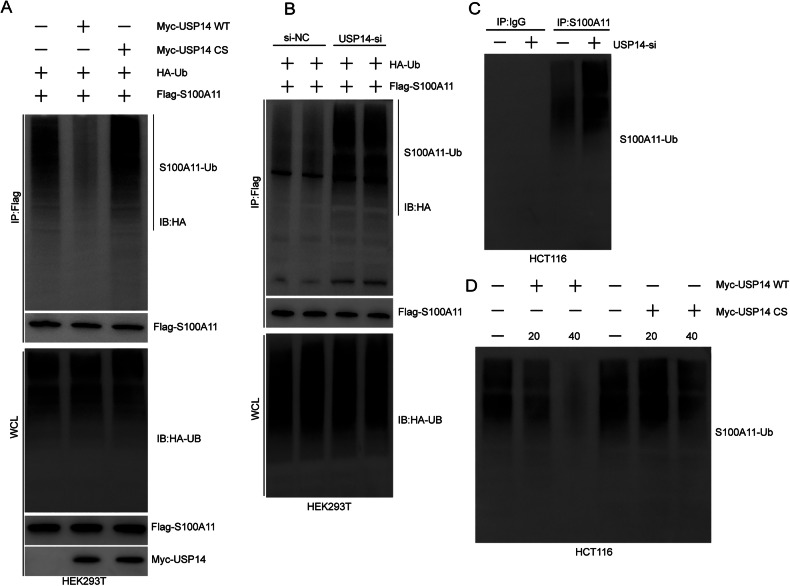
USP14 regulates S100A11 ubiquitination. **A** Co-immunoprecipitation in HEK293T cells showed decreased S100A11 ubiquitination with Myc-USP14 WT but not with Myc-USP14 CS. **B** Increased S100A11 ubiquitination upon USP14 knockdown in HEK293T cells. **C** Enhanced ubiquitination of S100A11 in HCT116 cells following USP14 knockdown. **D** Dose-dependent reduction of S100A11 ubiquitination with increasing amounts of Myc-USP14 WT in HCT116 cells.

The ubiquitination status of S100A11 was analysed to determine the regulatory role of USP14, revealing that overexpression of wild-type USP14 (Myc-USP14 WT) in HEK293T cells significantly reduced S100A11 ubiquitination (Fig. 6A). This suggests that USP14’s deubiquitinating activity is crucial for reducing S100A11 ubiquitination. Silencing USP14 in HEK293T cells increased S100A11 ubiquitination, further supporting the role of USP14 in modulating S100A11 stability through deubiquitination (Fig. 6B). USP14 knockdown in HCT116 cells enhanced S100A11 ubiquitination, confirming these findings in a CRC model (Fig. 6C). Additionally, there was a dose-dependent effect of USP14 on S100A11 ubiquitination with higher USP14 levels reducing ubiquitination, whereas no such change was evident following USP14 CS mutant plasmid transfection (Fig. 6D). Collectively, USP14 reduces S100A11 ubiquitination highlighting its role in stabilising S100A11 in CRC cells.

### USP14 acts as an oncogene through S100A11 in CRC cells

**Fig. 7:**
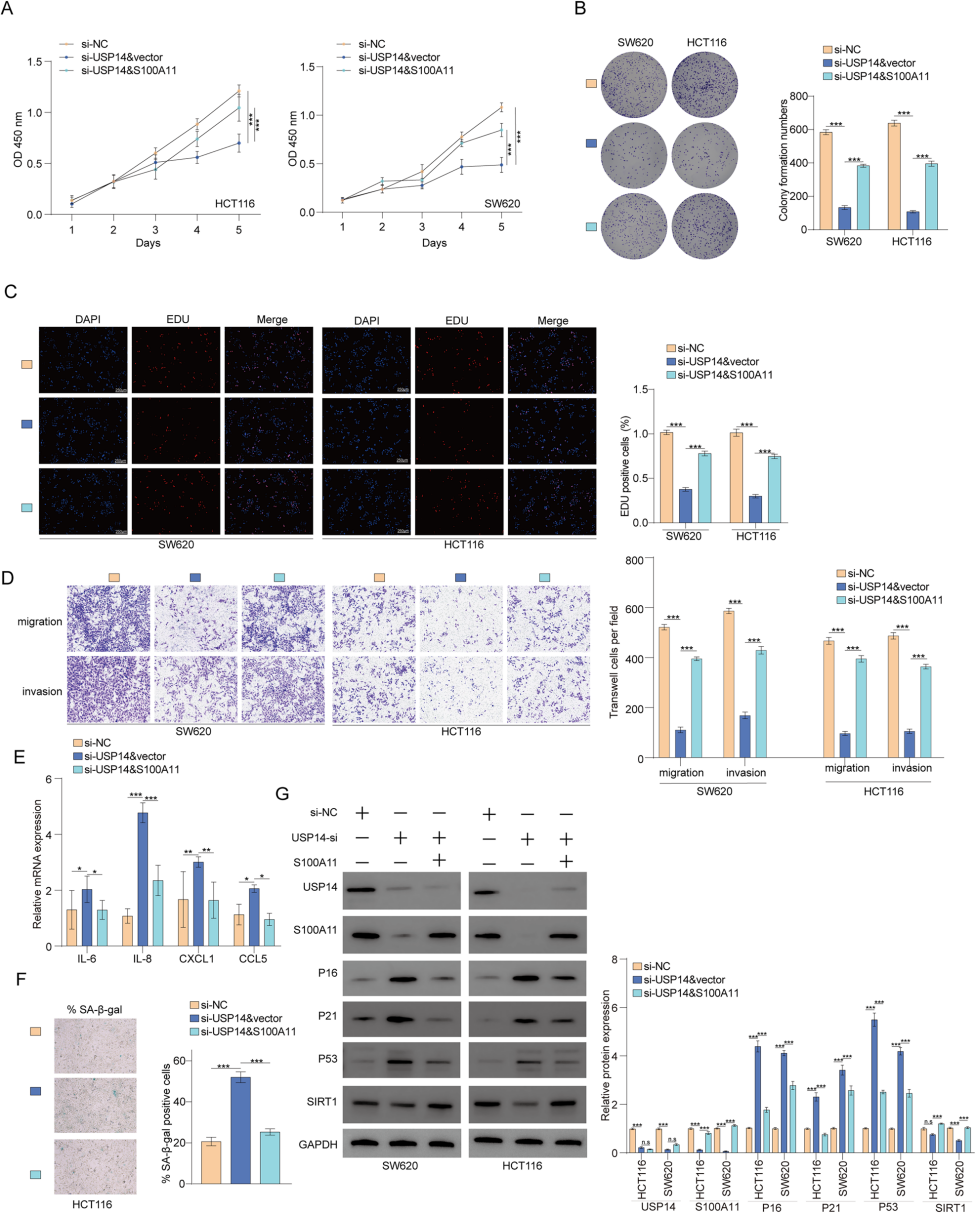
Effects of the USP14/S100A11 axis on colorectal cancer cell proliferation, migration, invasion, and senescence. **A** CCK-8 assays showed reduced proliferation with USP14 knockdown and partial rescue by S100A11 overexpression. **B** Colony formation assays indicated decreased colony numbers with USP14 knockdown. **C** EdU assays confirmed reduced proliferation upon USP14 knockdown. **D** Transwell assays showed inhibited migration and invasion with USP14 knockdown, partially reversed by S100A11. **E** RT-qPCR analysis of cytokine expression changes. **F** Senescence-associated β-galactosidase staining showed increased senescence with USP14 knockdown. **G** Western blot analysis of cell cycle and senescence-related proteins. *p < 0.05, **p < 0.01, ***p < 0.001.

The role of S100A11 in colorectal cancer cell proliferation, migration, and invasion was assessed in the context of USP14 knockdown. CCK-8 assays demonstrated that silencing USP14 significantly reduced HCT116 and SW620 cell proliferation, while S100A11 overexpression partially rescued this effect (Fig. 7A). Colony formation assays showed a similar trend with USP14 knockdown decreasing and partial restoration with S100A11 overexpression (Fig. 7B). EdU incorporation assays further confirmed USP14 knockdown reduced cell proliferation which was mitigated by S100A11 overexpression (Fig. 7C). Transwell assays revealed that USP14 knockdown significantly inhibited migration and invasion capabilities of both cell lines, whereas S100A11 overexpression partially reversed these effects (Fig. 7D). RT-qPCR analysis indicated that pro-inflammatory cytokine expression (IL-6, IL-8, CXCL1, CCL5) was modulated by USP14 and S100A11, knocking down USP14 enhances the expression of inflammatory cytokines, whereas overexpressing S100A11 mitigates this effect (Fig. 7E). Senescence-associated β-galactosidase staining demonstrated that USP14 knockdown increased cellular senescence which was alleviated by S100A11 overexpression (Fig. 7F). USP14 knockdown upregulated cell cycle inhibitors (P16, P21) and tumour suppressor P53, while SIRT1 levels decreased. S100A11 overexpression counteracted these changes (Fig. 7G). Transient S100A11 overexpression partially but significantly rescued the USP14 knockdown-induced tumour growth suppression (Supplementary Fig. S2). Collectively, USP14 knockdown inhibits CRC proliferation, migration, and invasion, while S100A11 overexpression partially reverses these effects.

### Expression of USP14 and S100A11 in CRC

**Fig. 8:**
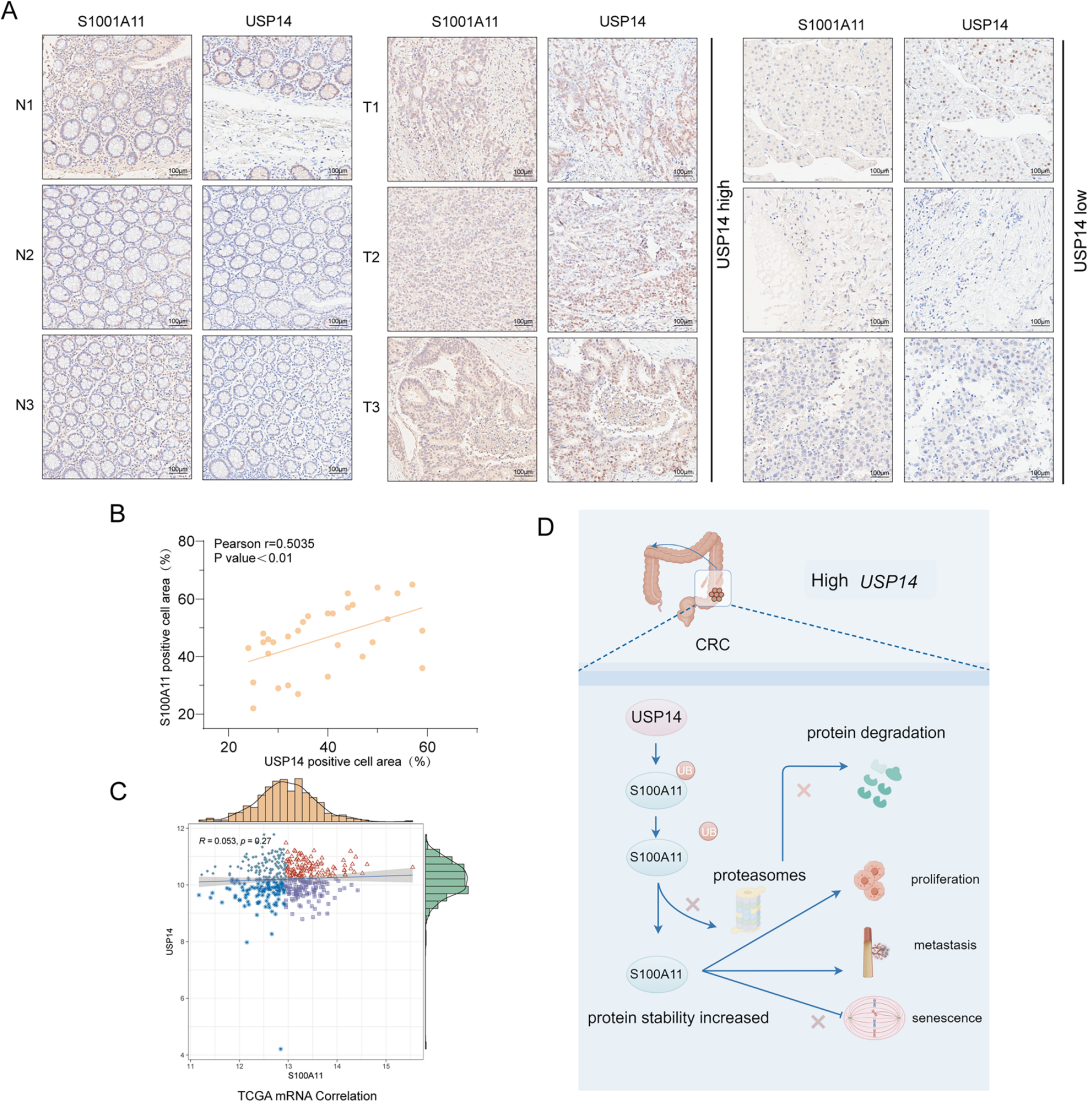
USP14 and S100A11 expression in colorectal cancer. **A** Immunohistochemical staining showed higher S100A11 and USP14 expression in CRC tissues compared to normal tissues. **B** Positive correlation between USP14 and S100A11 expression in CRC tissues. **C** TCGA data analysis showed no correlation between USP14 and S100A11 mRNA levels in CRC samples. **D** Schematic model of USP14’s role in regulating S100A11 stability and its impact on CRC progression.

Based on TCGA data and our experimental findings, there is no significant correlation between USP14 and S100A11 at the mRNA level (Fig. 8C). However, immunohistochemical analysis reveals a positive correlation at the protein level (Fig. 8B), indicating a post-transcriptional regulatory mechanism. The differential expression patterns observed in normal versus tumour tissues (Fig. 8A) further support the role of USP14 in modulating S100A11 protein stability, which may impact cellular processes such as proliferation and metastasis. It is hypothesised that high USP14 expression in CRC leads to decreased ubiquitination and degradation of S100A11, resulting in enhanced protein stability, thereby promoting CRC cell proliferation and metastasis, and reducing senescence, highlighting a potential oncogenic pathway(Fig. 8D).

## Discussion

The present study demonstrated the significant role of S100A11 in CRC progression, corroborating and extending previous observations of its involvement in various malignancies [14, 22–27]. Consistent with reports in other cancers, S100A11 is upregulated in CRC and is associated with poor survival outcomes. This aligns with the findings, which demonstrated that S100A11 promotes tumour growth and metastasis in breast cancer [28]. These findings across different tumour types highlight the potential of S100A11 as a universal marker of malignancy and a candidate for targeted therapy.

Cellular senescence has emerged as a double-edged sword in cancer biology [29]. While senescence prevents cell propagation with oncogenic mutations through stable cell cycle arrest, mediated by key tumour suppressors like p53, p21, and RB, it also contributes to tumour progression [30]. This paradoxical effect arises from the senescence-associated secretory phenotype (SASP) with the secretion of pro-inflammatory cytokines, growth factors, and proteases that promote tumour growth, angiogenesis, and immune evasion [31]. The role of senescence in tumour suppression is enhanced by external mechanisms. Senescent cells can induce senescence in neighbouring cells through both the SASP and direct cell interactions, thereby limiting the spread of nearby pre-malignant or fully malignant cells that have not yet undergone senescence. For example, the SASP factor TNFα can induce ROS-dependent apoptosis in human-derived cancer cell lines, while IL-6 initiates apoptosis in cultured neoplastic T lymphocytes [32, 33]. Consequently, senescence acts as an antitumour barrier in vivo, and the paracrine modulation of potential precursor lesions within the microenvironment further enhances its effectiveness.

The role of S100A11 in promoting cell proliferation and inhibiting senescence, as demonstrated in our study, provides a mechanistic insight into its contribution to cancer pathology. This anti-senescence effect is particularly significant given the established role of cellular senescence as a tumour suppressive mechanism. The inhibition of senescence by S100A11 could therefore represent a critical step in cancer progression, facilitating unchecked cell division and tumour growth.

The interaction between S100A11 and USP14 uncovered in our research adds a new layer to our understanding of CRC biology. USP14, a deubiquitinating enzyme, has garnered significant attention for its role in cancer progression, as it influences multiple cellular processes including protein degradation, cell cycle regulation, and apoptosis [17–20, 34, 35]. Elevated USP14 expression has been associated with poor prognosis in breast, colorectal, and pancreatic cancers [17, 34, 36]. The interaction between USP14 and S100A11 suggests a novel regulatory axis whereby USP14 may modulate the stability and function of S100A11, thus influencing CRC progression. This is supported by our findings that the modulation of USP14 levels affected S100A11-mediated cellular functions, indicating that targeting this interaction could be a viable therapeutic strategy. USP14 inhibitors have shown significant potential in cancer therapy, particularly for colorectal cancer, by suppressing tumour growth, enhancing chemotherapy and radiotherapy effectiveness, and overcoming drug resistance [37, 38]. Their role in modulating antitumour immune responses and serving as prognostic markers further underscores their clinical relevance [17]. These findings support the development of USP14 inhibitors as promising anticancer agents for CRC.

In conclusion, the present study not only confirms the pivotal role of S100A11 in CRC but also introduces the S100A11-USP14 interaction as a novel regulatory mechanism that could be exploited for therapeutic gains. Future studies should focus on developing inhibitors that specifically disrupt this interaction, potentially offering a new avenue for CRC treatment. Moreover, further research is needed to explore the broader implications of S100A11-mediated suppression of senescence in cancer progression and resistance to therapies.

## Materials and methods

### Clinical specimens

Formalin-fixed and paraffin-embedded CRC tumour samples were collected at the Second Affiliated Hospital of Wannan Medical College from 2018 to 2023. Written informed consent was provided by individuals donating CRC tissues. This study was approved by the Ethics Committee of the Second Affiliated Hospital of Wannan Medical College.

### Cell culture and treatment

The human colorectal cancer cell lines, HCT116, SW480, DLD1, and SW620, and the human cell lines FHC and HEK-293T were sourced from the Cell Bank at the Shanghai Institute of Cell Biology in Shanghai, China. These cells were maintained in 10% DMEM with 1% penicillin/streptomycin (Gibco, USA) at 37 °C in a 5% CO_2_ atmosphere. The cell lines were verified by short tandem repeat (STR) profiling and screened for mycoplasma contamination.

### Real-time RT-PCR

Total RNA was extracted using TRIzol reagent (Invitrogen, USA) and the concentration was determined by the NanoDrop ND2000 (Thermo Scientific, USA). The RNA was reverse transcribed using the HiScript II Q RT SuperMix (Vazyme, China) for qRT-PCR using the SYBR Green PCR kit (Vazyme, China) on the ABI StepOnePlus system (Applied Biosystems Inc., USA). Gene expression was quantified via the comparative cycle threshold (Ct) method (2−ΔCt) and normalised against β-actin. All experiments were conducted in triplicate. The primer sequences can be found in Supplementary Table S1.

### Immunoprecipitation and immunoblotting

The cells were lysed in RIPA lysis buffer supplemented with a complete protease inhibitor cocktail (Roche), centrifuged at 4 °C and precleared with protein A/G agarose beads (Santa Cruz, sc-2003) at 4 °C for 1 h. The samples were centrifuged at 1000 × *g* for 5 min at 4 °C and the resultant cell lysates were transferred into new tubes for IP with the specified antibodies and incubated overnight at 4 °C. Subsequently, the samples were incubated with 50 µl of protein A/G agarose beads at 4 °C. The immunoprecipitates were obtained by centrifugation and washed with RIPA buffer before resuspension in electrophoresis sample buffer. The protein samples were transferred to polyvinylidene difluoride membranes (Millipore) and the membranes were blocked for 1 h at room temperature using 0.1% Tween-20 in Tris-buffered saline (TBS) containing 5% skimmed milk. The membranes were incubated with the specified primary antibodies, followed by HRP-conjugated secondary antibodies and the protein bands were visualised on an Amersham Imager 600 system (GE Healthcare).

### Immunohistochemistry

IHC staining was performed on sections of tumours derived from human tumour and xenograft mouse tissues. The tissue sections underwent de-paraffinization and rehydration, after which they were immersed in 3% H_2_O_2_ for a duration of 10 min. Antigen retrieval was achieved by treating the sections with citrate buffer (pH 6.0) at a temperature of 97 °C, allowing them to cool naturally to room temperature afterward. Subsequently, the sections were blocked using 10% BSA, and the primary antibodies were applied to separate slides, followed by an overnight incubation at 4 °C. The antibodies are detailed in Supplementary Table S2. Two pathologists blinded to the patients’ clinical characteristics evaluated the samples independently focusing on the staining intensity and distribution. The scoring for the percentage of positive cells ranged from 0 to 4 (0 for <10%, 1 for 10–30%, 2 for 30–50%, 3 for 50–80%, 4 for 80–100%) and the staining intensity was scored from 0 to 3 (0 for no staining, 1 for weak, 2 for moderate, and 3 for strong). The final immune response score was determined by multiplying the staining intensity score by the percentage of positive cells.

### Ubiquitination assay

The CRC cells were co-transfected with the plasmids Myc-USP14, HA-UB, Flag-S100A11, or Flag-S100A11-mut and then lysed and processed according to the immunoprecipitation protocol.

### ELISA detection

Cell supernatants were collected following 48-h culture, centrifuged at 300 × *g* for 5 min to remove debris, and stored at −80 °C. Cytokine levels (IL-6, IL-8, CXCL1 and CCL5) were measured using specific ELISA kits (CUSABIO, China) according to the manufacturer’s instructions. Briefly, 50 μL of standards/samples were loaded onto antibody-precoated plates (duplicate wells), incubated for 1 h at RT, followed by sequential 30-min incubations with biotinylated detection antibodies and streptavidin-HRP (1:200). Absorbance (450/540 nm) was quantified via BioTek Synergy H1.

### Cell proliferation assays

Cell proliferation was assessed by counting the number of cell clones and evaluating their viability using the CCK-8 kit (Cell Counting Kit-8, Beyotime) according to the manufacturer’s protocol. For colony formation, 3000 cells were seeded per well in a 6-well plate and incubated for 2 weeks before the fixed cells were stained with crystal violet and counted.

### Flow cytometry analysis

Flow cytometry analysis was employed to assess apoptosis using Annexin V/PI staining. Briefly, CRC cells were harvested and washed twice with cold PBS, followed by resuspension in 1× binding buffer. Subsequently, 100 µL of the cell suspension was transferred to a 5 mL culture tube, and 5 µL of FITC-conjugated Annexin V and 5 µL of propidium iodide (PI) were added. The cells were gently vortexed and incubated for 15 min at room temperature in the dark. After incubation, 400 µL of 1× binding buffer was added to each tube. Samples were analysed using a flow cytometer within 1 h, and data were processed to determine the percentage of apoptotic cells.

### In vivo experiments

The Institutional Animal Care and Use Committee of The Second Affiliated Hospital of Wannan Medical College approved the animal experiments. The mouse model of tumour formation was established by subcutaneous injection of 1 × 10^6^ cells into the left flank of male nude mice at 5 weeks of age (VitalRiver Laboratory Animal Co., Ltd., Beijing, China). The tumour volume was calculated using the formula: volume = width² × length × 0.5. After 4 weeks, all experimental mice were euthanised to harvest the tumours.

### Transwell migration and invasion assays

Cell migration was evaluated by utilising a 24-well transwell chamber featuring an 8.0 μm pore size filter (Corning, Canton, NY). The cells were suspended in serum-free medium and 6.0 × 10^4^ cells were seeded into the upper chamber, with 600 μl of 10% serum medium in the lower chamber. After incubation for 24 h at 37 °C, the chambers were gently washed twice with PBS, fixed with methanol, and subsequently stained with crystal violet (Solarbio, Beijing, China). Migrated cells were counted at a magnification of 200× in three fields per filter. The same procedure was followed for the invasion assay, except the wells were pre-coated with Matrigel (Corning, Canton, NY, USA).

### Senescence assay

Senescence was assessed according to the protocol described by Debacq-Chainiaux et. al [39]. The cells were fixed in 5% formaldehyde and then incubated with a staining solution (Sigma) containing 1 mg/ml of 5-bromo-4-chloro-3-indolyl β-d-galactosidase (X-Gal, Invitrogen) for 24 h at 37 °C before the distinct blue cells were counted at a magnification of 20× and the counts were normalised to the total cell number.

### Statistical analysis

Statistical analysis was performed using GraphPad Prism version 8.0 and SPSS version 21.0. Data are presented as the mean ± standard deviation (SD) and analysed with the Student’s t-test and Spearman’s correlation analysis as appropriate. A *p-*value ≤ 0.05 was considered statistically significant.

## Supplementary information

Figure S1

Figure S2

Supplementary Figure Legends

Supplementary Table S1

Supplementary Table S2

WB ORIN

## Data Availability

The datasets used and/or analysed during the current study are available from the corresponding author on reasonable request.
