# Supplementary figures and images for: USP14/S100A11 axis promote colorectal cancer progression by inhibiting cell senescence
Source: Cell Death Dis. 2025 May 15;16(1):384. doi: 10.1038/s41419-025-07724-8 (PMC12081677; doi:10.1038/s41419-025-07724-8)

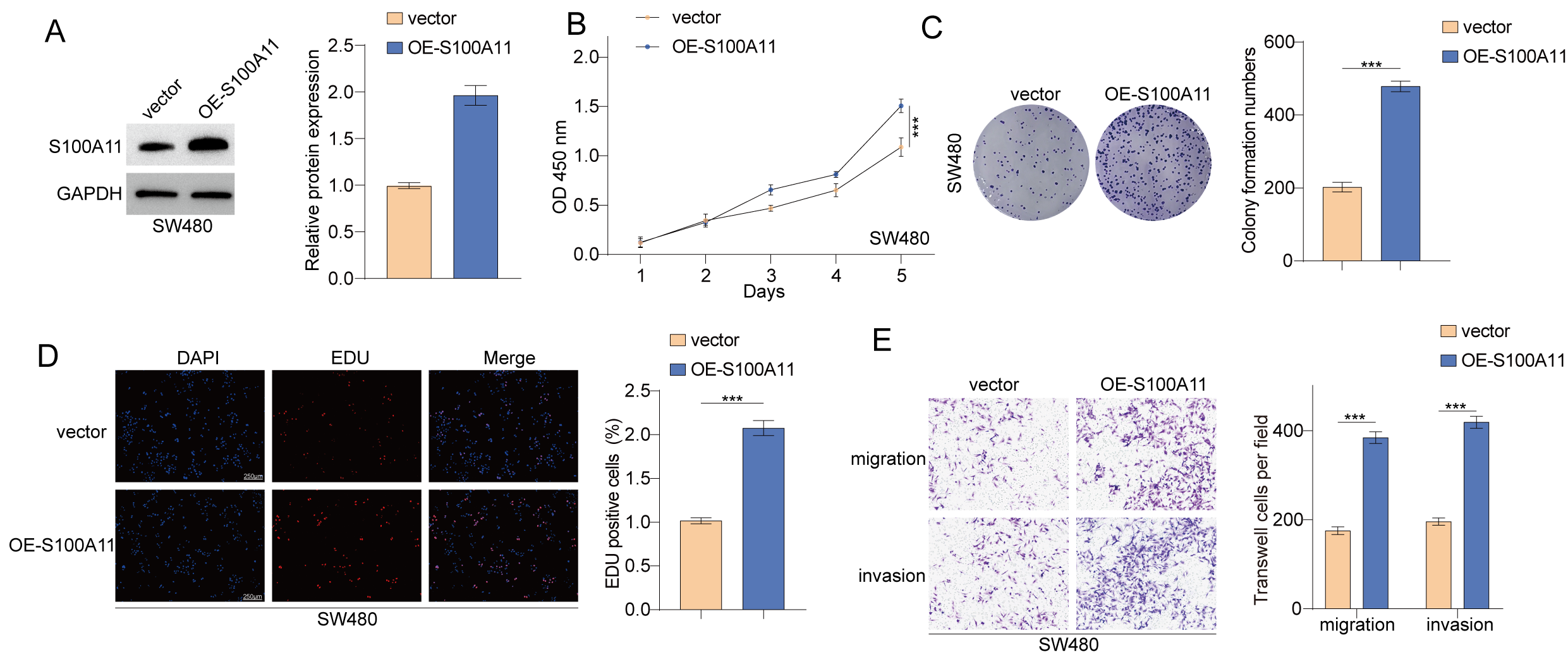

Supplement: Supplementary file 1 — Figure S1 [file 41419_2025_7724_MOESM1_ESM.tif]

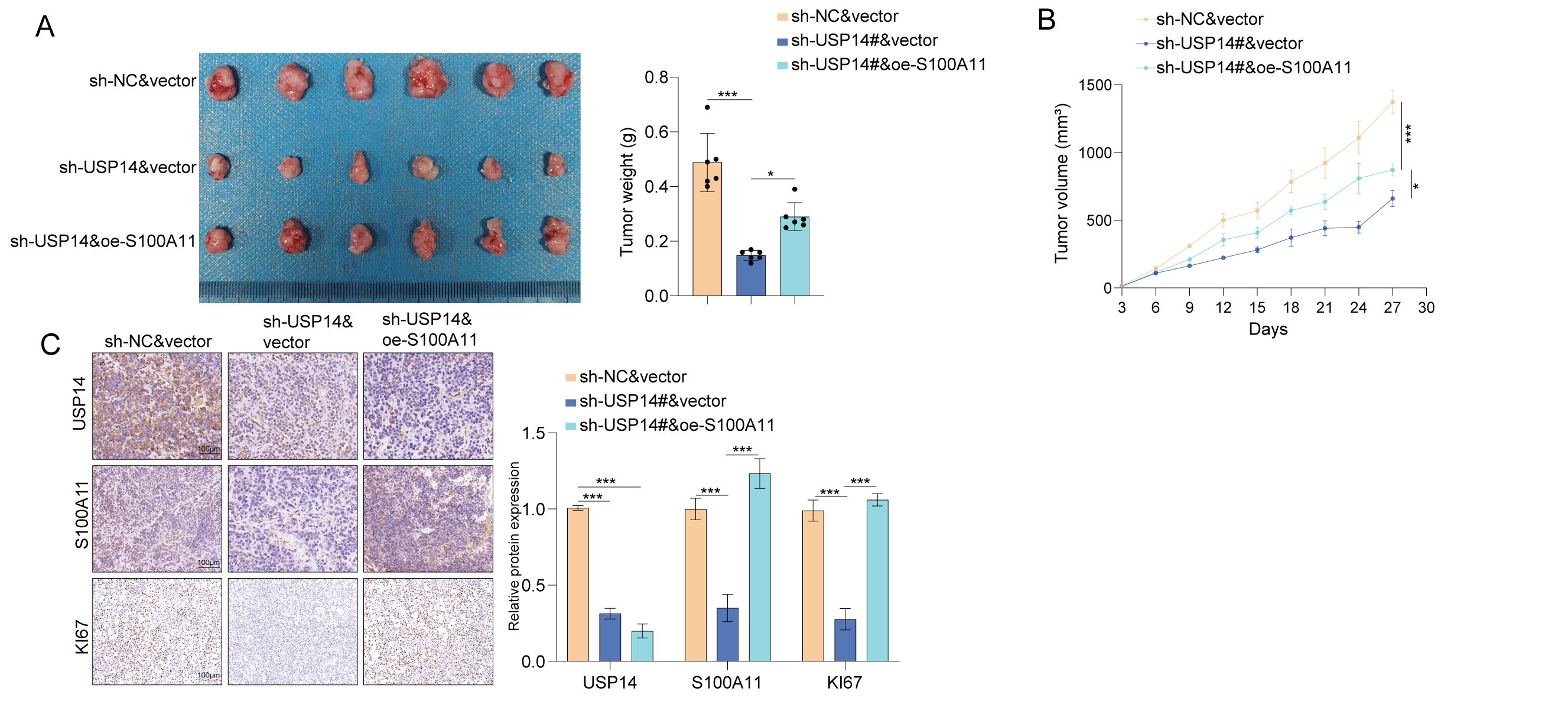

Supplement: Supplementary file 2 — Figure S2 [file 41419_2025_7724_MOESM2_ESM.tif]

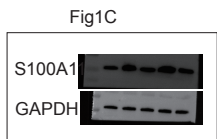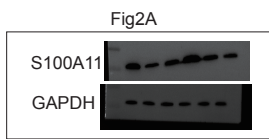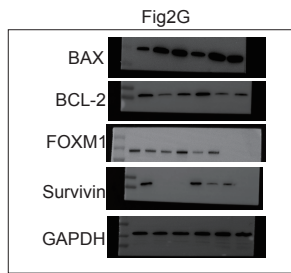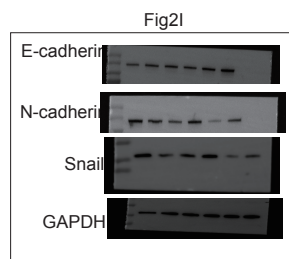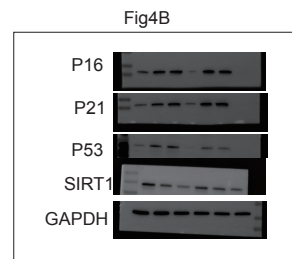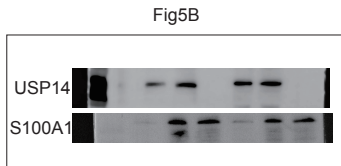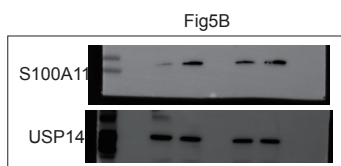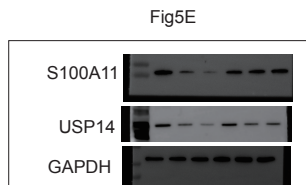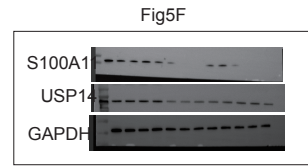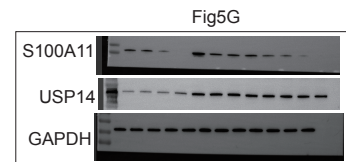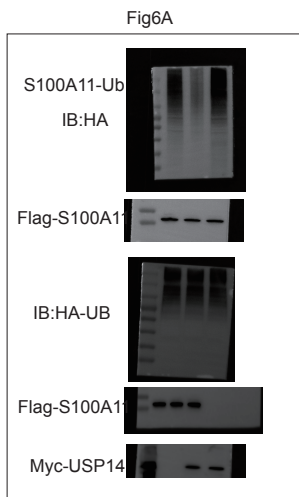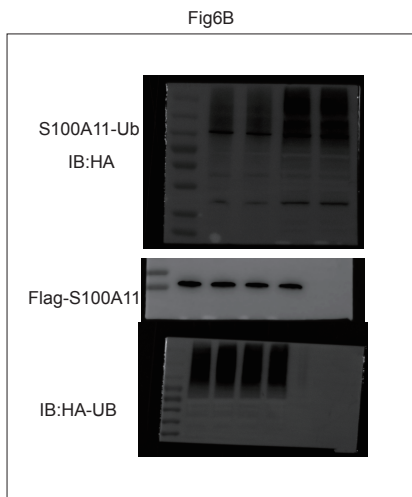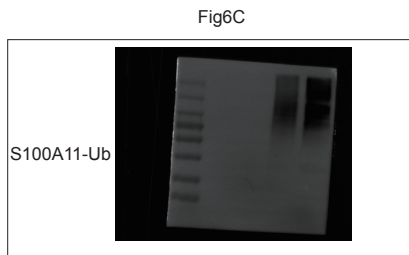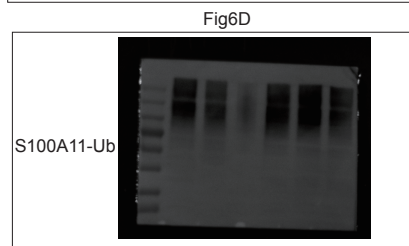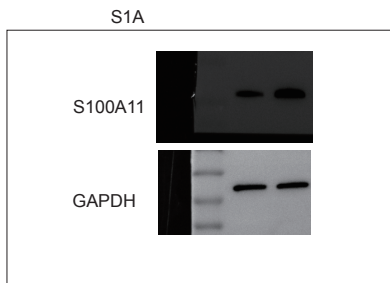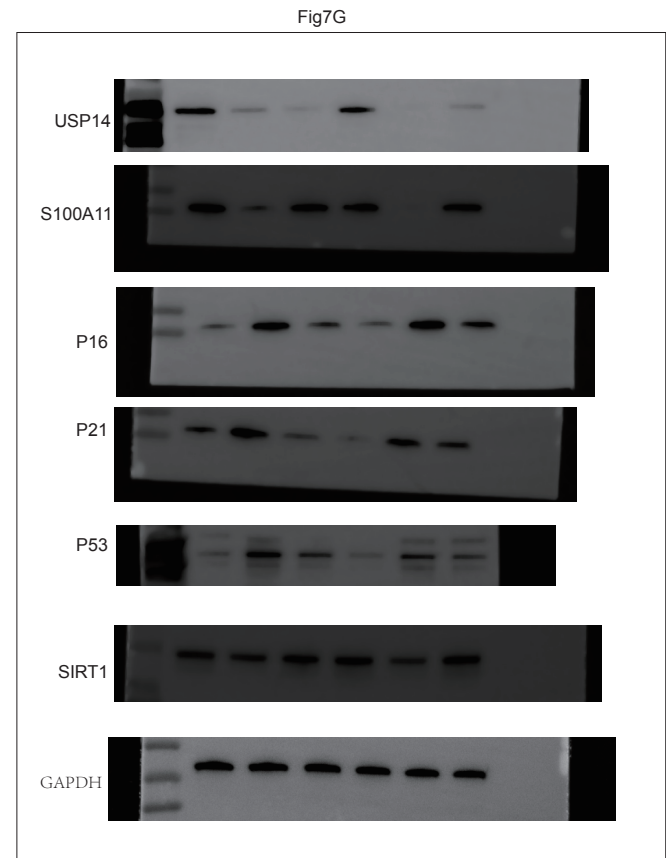

Supplement: Supplementary file 6 — WB ORIN [file 41419_2025_7724_MOESM6_ESM.pdf]
