# Supplementary material for: USP14/S100A11 axis promote colorectal cancer progression by inhibiting cell senescence
Source: Cell Death Dis. 2025 May 15;16(1):384. doi: 10.1038/s41419-025-07724-8 (PMC12081677; doi:10.1038/s41419-025-07724-8)
Supplement: Supplementary file 3 — Supplementary Figure Legends [file 41419_2025_7724_MOESM3_ESM.docx]

Supplementary Figure legends

**Supplementary Figure S1.** S100A11 overexpression promotes CRC cancer cell proliferation and invasion. SW480 was transfected with negative control plasmid (vector) or pcDNA3.1-S100A11 plasmid (S100A11). (A) The relative expression levels of S100A11 were analyzed by western blot. Cell proliferation capability of SW480 transfected with vector or S100A11 was determined by CCK-8 assay (B), colony formation assay (C) and EdU assay (D); Scale bar = 250 μm. (E) Transwell experiment was performed to analyze the cell invasion capability of SW480 cells transfected with vector or S100A11. The results are presented as the mean ± SD. *P <0.05, **P <0.01, ***P <0.001.

**Supplementary Figure S2**. (A)Effects of USP14 knockdown and S100A11 overexpression on CRC tumor weight. Representative images of tumors and a bar graph showing significantly reduced tumor weight in the sh-USP14#&vector group compared to sh-NC&vector. The combination group (sh-USP14#&oe-S100A11) shows partial restoration of tumor weight (*p<0.05). (B) Tumor volume measurements over time. The results are presented as the mean ± SD. *P <0.05, **P <0.01, ***P <0.001. (C) Immunohistochemical analysis of USP14, S100A11, and Ki67 expression in tumor tissues from each group. Representative images are shown with quantification of relative protein expression levels. Scale bars represent 100 μm. Statistical significance is indicated (***p<0.001).
