# Supplementary material for: USP14/S100A11 axis promote colorectal cancer progression by inhibiting cell senescence
Source: Cell Death Dis. 2025 May 15;16(1):384. doi: 10.1038/s41419-025-07724-8 (PMC12081677; doi:10.1038/s41419-025-07724-8)
Supplement: Supplementary file 4 — Supplementary Table S1 [file 41419_2025_7724_MOESM4_ESM.docx]

| **Name** | **Supplier** | **Catalog number** | **APPLICATION** |
| --- | --- | --- | --- |
| S100A11 | Proteintech | 10237-1-AP | WB/IHC/IF |
| GAPDH | Proteintech | 10494-1-AP | WB |
| BAX | Proteintech | 50599-2-Ig | WB |
| BCL-2 | Proteintech | 26593-1-AP | WB |
| FOXM1 | Proteintech | 13147-1-AP | WB |
| Survivin | Proteintech | 110508-1-AP | WB |
| N-cadherin | Abclonal | A19083 | WB |
| E-cadherin | Abclonal | A3044 | WB |
| snail | abcam | ab216347 | WB |
| USP14 | Proteintech | 14517-1-AP | WB/IHC/IF |
| HA-tag | Proteintech | 51064-2-AP | WB |
| Flag-tag | Proteintech | 20543-1-AP | WB |
| Myc-tag | Proteintech | 60003-2-Ig | WB |
| Ki-67 | Abclonal | A11390 | IHC |
| P16 | Proteintech | 10883-1-AP | WB |
| P21 | Proteintech | 10355-1-AP | WB |
| P53 | Proteintech | 10442-1-AP | WB |
| SIRT1 | Proteintech | 13161-1-AP | WB |
