# Supplementary material for: USP14/S100A11 axis promote colorectal cancer progression by inhibiting cell senescence
Source: Cell Death Dis. 2025 May 15;16(1):384. doi: 10.1038/s41419-025-07724-8 (PMC12081677; doi:10.1038/s41419-025-07724-8)
Supplement: Supplementary file 5 — Supplementary Table S2 [file 41419_2025_7724_MOESM5_ESM.docx]

**Table S2. Primer information for qPCR analysis.**

| **Primer name** | **Accession number** | **GC content (%)** | **Tm (°C)** | **Annealing**  **Temperature (°C)** | **Primer sequence** |
| --- | --- | --- | --- | --- | --- |
| S100A11 Divergent primer Forward | NM_016740 | 52.38 | 62.9 | 60 | GCGGGAAGGATGGAAACAACA |
| S100A11 Divergent primer Reverse | NM_016740 | 52.38 | 62.4 |  | TCATCATGCGGTCAAGGACAC |
| GAPDH Divergent primer Forward | NM_001357943 | 68.42 | 68.7 | 60 | CATGGCCCACATGGCCTCC |
| GAPDH Divergent primer Reverse | NM_001357943 | 55.56 | 62.3 |  | GTTCTCAGCCTTGACGGT |
| IL-8 Forward | NM_000584 | 40.9 | 60.1 | 60 | TTTTGCCAAGGAGTGCTAAAGA |
| IL-8 Reverse | NM_000584 | 52.38 | 62.5 |  | AACCCTCTGCACCCAGTTTTC |
| IL-6 Forward | NM_000600 | 43.47 | 60.2 | 60 | ACTCACCTCTTCAGAACGAATTG |
| IL-6 Reverse | NM_000600 | 47.82 | 61.8 |  | CCATCTTTGGAAGGTTCAGGTTG |
| CXCL1 Forward | NM_008176 | 55.00 | 62.7 | 60 | ACTGCACCCAAACCGAAGTC |
| CXCL1 Reverse | NM_008176 | 45.45 | 61.3 |  | TGGGGACACCTTTTAGCATCTT |
| CCL5 Forward | NM_002985 | 55.00 | 60.9 | 60 | CCAGCAGTCGTCTTTGTCAC |
| CCL5 Reverse | NM_002985 | 50.00 | 62.3 |  | CTCTGGGTTGGCACACACTT |
